# Diagnostic accuracy of basal TSH determinations based on the intravenous TRH stimulation test: An evaluation of 2570 tests and comparison with the literature

**DOI:** 10.1186/1472-6823-7-5

**Published:** 2007-08-02

**Authors:** Helga Moncayo, Otto Dapunt, Roy Moncayo

**Affiliations:** 1 Department of Obstetrics and Gynecology, University of Innsbruck, Anichstrasse 35, A-6020 Innsbruck, Austria; 2 Department of Nuclear Medicine, Medical University Innsbruck, Austria; 3 WOMED, Karl-Kapferer-Strasse 5, 6020 Innsbruck, Austria

## Abstract

**Background:**

Basal TSH levels reflect the metabolic status of thyroid function, however the definition and interpretation of the basal levels of TSH is a matter of controversial debate. The aim of this study was to evaluate basal TSH levels in relation to the physiological response to i.v. TRH stimulation.

**Methods:**

A series of 2570 women attending a specialized endocrine unit were evaluated. A standardized i.v. TRH stimulation test was carried out by applying 200 μg of TRH. TSH levels were measured both in the basal and the 30 minute blood sample. The normal response to TRH stimulation had been previously determined to be an absolute value lying between 2.5 and 20 mIU/l. Both TSH values were analyzed by cross tabulation. In addition the results were compared to reference values taken from the literature.

**Results:**

Basal TSH values were within the normal range (0.3 to 3.5 mIU/l) in 91,5% of cases, diminished in 3,8% and elevated in 4.7%. Based on the response to TRH, 82.4% were considered euthyroid, 3.3% were latent hyperthyroid, and 14.3% were latent hypothyroid. Combining the data on basal and stimulated TSH levels, latent hypothyroidism was found in the following proportions for different TSH levels: 5.4% for TSH < 2.0 mIU/l, 30.2% for TSH between 2.0 and 3.0 mIU/l, 65,5% for TSH between 3.0 and 3.50 mIU/l, 87.5% for TSH between 3.5 and 4.0 mIU/l, and 88.2% for TSH between 4 and 5 mIU/l. The use of an upper normal range for TSH of 2.5 mIU/l, as recommended in the literature, misclassified 7.7% of euthyroid cases.

**Conclusion:**

Our analysis strategy allows us to delineate the predictive value of basal TSH levels in relation to latent hypothyroidism. A grey area can be identified for values between 3.0 and 3.5 mIU/l.

## Background

Elevated levels of TSH are the hallmark of decreased thyroid function. In order to correctly identify these patients it is imperative to have a clear definition of the upper reference range for basal TSH. Patients whose TSH lies in the upper reference range might appear to have minimal thyroid deficiency. Although this might appear to be an easy task, the definition of the upper reference range for TSH has been matter of controversial debate [1-5]. Reported reference values for the upper range of basal TSH vary between 2.12 and 5.95 mIU/l [6-21] (Table 1). In the majority of studies, the reference range for TSH has been defined by statistical analysis (95% confidence interval) of log transformed data. An alternative approach is to rely on the physiological response of TSH to TRH stimulation [22] taking the absolute TSH values 30 minutes after TRH stimulation as the classification criteria [17,23,24].

**Table 1:** Comparison of TSH reference levels from the literature

**Author**	**Year**	**n**	**Population Age in years**	**TSH levels**
De Rosa [6]	1996	259	12 – 82	0.3 – 3.32
Taimela [7]	1997	262	23 – 69	0.6 – 4.3
Bjoro [8]	2000	19327 f 9754 m	20–80	0.49 – 5.70 f 0.56 – 4.60 m
Steinmetz [9]	2000	1348	45–70	0.43 – 3.71
Hollowell [10]	2002	13344	12 – 80	0,45 – 4,12
Hubl [11]	2002	1030	18 – 90	0.19 – 4.25
Hübner [12]	2002	43	15–18 (study subgroup)	0.56 – 4.53
Gonzalez-Sagrado [13]	2004	304	12–94	0.51 – 5.95
Jensen [14]	2004	1512	17 – 66	0.58 – 4.07
D'Herbomez [15]	2005	710	18 – 65	0.35 – 3.48
Kratzsch [16]	2005	870	18 – 68	0.3 – 3.63
Moncayo [17]	2005	14981	20–89	0.3 – 3.5
Völzke [18]	2005	1488	20–79	0.25 – 2.12
Zöphel [19]	2005	1442	Unkown	0.3 – 3.35
Dhatt [20]	2006	959	16 – 75	0.3 – 4.32
O'Leary [21]	2006	2026	29 – 70	0.4 – 4.0
Total number of observations		69659		

Within the setting of a fertility unit, the exclusion or identification of causes of infertility requires a broad diagnostic approach. In a recent review by Poppe, Velkeniers, and Glinoer, special emphasis was put on the evaluation of thyroid function [25]. Referring to thyroid function tests, the authors commented the apparent discrepancy between basal and TRH-stimulated TSH levels used to detect subclinical hypothyroidism (Table 1 in [25]). The aim of this retrospective study was to describe in more detail the relation of basal TSH levels to the TRH-stimulated levels and their validity for the detection of latent hypothyroidism. Data analysis was also carried out in comparison with criteria reported in the literature.

## Methods

The laboratory methods used have been described elsewhere [17]. A total of 2570 women attending the out-patient unit for Reproductive Endocrinology at the University of Innsbruck were studied (mean age 31 years, min. 21 years, max. 69 years). All patients were ambulatory and did not present any severe disease nor were taking any medication that could interfere with thyroid function tests. Ninety percent of patients were consulting the service due to irregularities of the menstrual cycle, or infertility. Ten percent of cases corresponded to menopausal women. Patients with known thyroid disease were excluded. Informed consent was obtained according to the Declaration of Helsinki. The study was approved by the institutional Ethics Committee. The TRH test was carried out by i.v. application of 200 μg of TRH. Both a basal and a 30 minute blood sample were obtained. In the first sample, both thyroid hormones (fT3 and fT4) and TSH were determined; in the second one only TSH was measured. Antibody levels were not determined. The reference values for basal TSH had been previously evaluated based on ROC analysis and lied between 0.3 to 3.5 mIU/l [17]. These reference values will be called I-TSH in further comparisons. An absolute TSH increase between 2.5 and 20 mIU/l following TRH stimulation was defined as normal [17]. The definition of these levels comes from a detailed internal evaluation of both clinical and laboratory data of 2870 patients being investigated at the out-patient unit of the Department of Nuclear Medicine, University of Innsbruck in 1993. The data from these patients was analyzed in relation to age, gender, iodine excretion, scintigraphy, sonography, and thyroid function tests (data not shown). These criteria were also used in a later analysis of 12.838 subjects done 2003 [17]. Altogether a total of 15708 subjects provide the foundation for the present analysis.

The reference ranges from the literature were taken from Zöphel [19] and Baloch [26]. These will be referred to as Z-TSH and B-TSH. A normal measured Z-TSH lies between 0.3 to 3.35 mIU/l, while a normal B-TSH has been proposed to be between 0.4 to 2.5 mIU/l.

### Data Analysis

The laboratory results were classified according to the criteria presented by Dayan [27]. Statistical analysis (one way ANOVA) was done with SPSS version 12. A p-value less than 0.01 was called significant.

## Results

### Analysis of fT3 and fT4 and of basal TSH levels

Stratification of data according to age groups did not reveal any age-dependency of thyroid function parameters (fT3, fT4, or TSH; one way ANOVA, data not shown). The classification of cases according to the levels of the free thyroid hormone showed 86,65% of cases to lie within the normal range, 2.3% lied above and 11.05% below. The proportion of patients having a normal basal TSH according to the 3 evaluation criteria was: 91.52% for I-TSH, 90.66 for Z-TSH and 83.77 for B-TSH, respectively. The lower number of euthyroid cases using the B-TSH criteria was accompanied by a 7.76% increase in the proportion of cases classified as having elevated TSH levels (Table 2).

**Table 2:** Categorized results according to 4 classification criteria for normal: 1) basal TSH according to Innsbruck criteria (I-TSH), 2) TRH stimulated TSH levels (s-TSH), 3) basal TSH according to Zöphel (Z-TSH), 4) basal TSH according to Baloch (B-TSH). The figures in the table correspond to the percentage of cases from the total that fall within a specific category.

I-TSH	%	s-TSH	%	Z-TSH	%	B-TSH	%
< 0.3	3,81	< 2,5	3,35	< 0.3	3,81	< 0.3	3,81
0.3 – 3.5	91,52	< 19,9	82,37	0.3 – 3.35	90,66	0.3 – 2.5	83,77
> 3.5	4,67	>= 20	14,28	> 3.35	5,53	> 2.5	12,41

A cross tabulation analysis of both free thyroid hormones and basal TSH (Table 3) showed that the normal ranges for the I-TSH and Z-TSH criteria corresponded to 80 and 79,34% of the subjects who also had normal thyroid hormone (TH) levels. In contrast to this, the proposed B-TSH criteria recognized only 73,46% of cases with normal TH levels as having a normal TSH. Again, the proposed B-TSH criteria classified twice as many patients as the I-TSH and Z-TSH criteria as hypothyroid/latent hypothyroid.

**Table 3:** Cross tabulation for the categorized results according to free thyroid hormones and the TSH criteria. The figures in the table correspond to the percentage of cases from the total that fall within a specific category

			Thyroid Hormones		
			
	b TSH		low	normal	elevated
TSH Innsbruck	< 0.3	n	8	71	19
		% total	0,31	2,76	0,74
	0.3 – 3.5	n	257	2056	39
		% total	10	80	1,52
	> 3.5	n	19	100	1
		% total	0,74	3,89	0,04

TSH Zöphel	< 0.3	n	8	71	19
		% total	0,31	2,76	0,74
	0.3 – 3.35	n	253	2039	38
		% total	9,84	79,34	1,48
	> 3.35	n	23	117	2
		% total	0,89	4,55	0,08

TSHBaloch	< 0.3	n	8	71	19
		% total	0,31	2,76	0,74
	0.3 – 2.5	n	231	1888	34
		% total	8,99	73,46	1,32
	> 2.5	n	45	268	6
		% total	1,75	10,43	0,23

	s TSH		low	normal	elevated

Groups according to stimulated TSH values	< 2,5	n	7	60	19
		% total	0,27	2,33	0,74
	< 19,9	n	224	1857	36
		% total	8,72	72,26	1,4
	>= 20	n	53	310	4
		% total	2,06	12,06	0,16

### Analysis of the stimulated TSH levels

**Figure 1:**
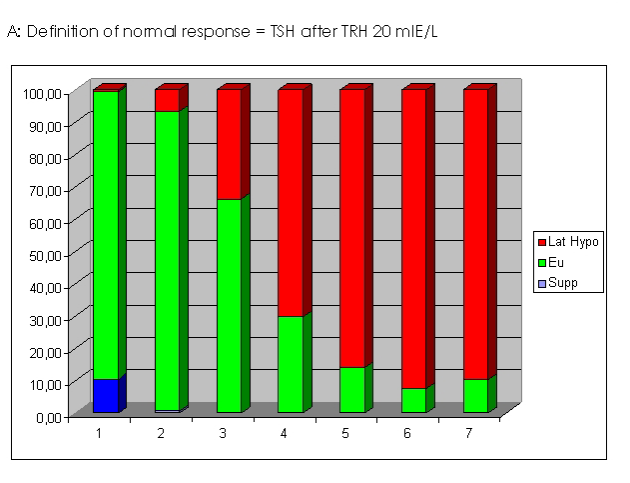
The diagram shows the response of TSH to TRH stimulation. The x-axis shows increasing TSH levels. The Y-axis shows that proportion of patients having a suppressed TSH response (blue), a normal response (green) or an exagerated response (red), i.e. latent hypothyroidism.

When the normal TSH response to TRH was compared to basal TSH levels, 80.81% of cases were called normal according to I-TSH, 80.54% for Z-TSH and 76.79% for B-TSH. Comparing both TSH results, i.e. basal and stimulated, an increasingly significant number of latent hypothyroid patients could be found when basal TSH was between 3 and 4. Latent hypothyroidism, defined as an elevated TSH after TRH, was found in the following proportions for different TSH levels: 5.4% for TSH < 2.0 mIU/l, 30.2% for TSH between 2.0 and 3.0 mIU/l, 65,5% for TSH between 3.0 and 3.50 mIU/l, 87.5% for TSH between 3.5 and 4.0 mIU/l, and 88.2% for TSH between 4 and 5 mIU/l (Figure 1).

## Discussion

The environment of this study reflects a common situation encountered in modern medicine, i.e. a reference laboratory will only have a blood sample for the biochemical evaluation of the patient. For this reason, the clinical validity of any test has to be evaluated in relation to the physiology of the system being investigated. In the case of thyroid physiology, the TRH test provides a physiological insight of the metabolic state [22]. For this reason we used the information provided by the TRH stimulation test for the evaluation of basal TSH levels in relation to the identification of latent and/or overt hypothyroidism. The normal range for TSH after TRH stimulation allowed us a similar categorical classification of patients as done with the values of free TH, thus underscoring the physiological meaning of the TRH test. On the other hand, due to the use of sensitive TSH assays, the identification of hyperthyroidism does not represent a problem, although in some cases the determination of TSH alone can be deceiving [27].

Recently, the proposition put forth by Baloch et al. [26] to define the reference levels of TSH based on a preselected (biased ?) population of healthy subjects has been investigated by Zöphel et al. [19]. In spite of adhering to Baloch's theoretical postulate, they were not able to find a difference in the reference levels for TSH using a pre-selected or an unselected population [19]. Their result shows, that pre-selection of normals based on thyroid antibody titers and other parameters is not a requisite for the definition of TSH levels. These conclusions apply to our study. Interestingly the normal range defined by Zöphel shows a good correlation with our results, those of Baloch do not.

Several lines of evidence have appeared recently relating altered body function in the presence of sub clinical hypothyroidism making it relevant to be able to identify such patients [28-32]. Since our study was purely diagnostic, we can not compare our results to approaches that try to analyze the cost-effectiveness of treating latent hypothyroidism. On the other hand, the original setting of this evaluation was predominantly related to problems related to fertility, i.e. the field of reproductive endocrinology. Using a similar approach as ours, Raber et al. have been able to show that infertility can be positively influenced by thyroid hormone supplementation when latent hypothyroidism is present [24]. Poppe et al. have recently reviewed the relation between thyroid disease and reproductive function in females. They have pointed out that several strategies are used for the definition of the upper level for TSH in order to detect subclinical hypothyroidism [25]. In their analysis, they suggest that data taken from TRH-stimulation tests appear to be more sensitive than other methods for defining normal TSH levels [25]. This situation is of outmost importance for pregnancy outcome [33]. The use of risk population definitions based on the presence of thyroid antibodies will miss a significant number of women presenting overt/subclinical hypothyroidism during early pregnancy [34]. Positive thyroid antibodies in pregnant women might be a reflection of a Se deficiency state which requires supplementation in order to prevent thyroid disease [35]. In our experience Se supplementation can also be beneficial in cases of incipient thyroid disease [36] leading to a normalization of both thyroid function parameters as well as of thyroid function.

## Conclusion

Our data analysis strategy supports a normal reference range for TSH of 0.3 to 3.5 mIU/l. Either a clinical and laboratory control or a TRH test can be recommended for patients with basal TSH values lying between 3.0 and 3.5 mIU/l in order to rule out latent hypothyroidism.

## Abbreviations

TSH: thyroid stimulating hormone

## Competing interests

The author(s) declare that they have no competing interests.

## Authors' contributions

OD and HE carried out the clinical work with the patients. RM created the data based and carried out the statistical analyses.

## Pre-publication history

The pre-publication history for this paper can be accessed here:


